# Cross-sectional survey evaluating Text4Mood: mobile health program to reduce psychological treatment gap in mental healthcare in Alberta through daily supportive text messages

**DOI:** 10.1186/s12888-016-1104-2

**Published:** 2016-11-08

**Authors:** Vincent I. O. Agyapong, Kelly Mrklas, Michal Juhás, Joy Omeje, Arto Ohinmaa, Serdar M. Dursun, Andrew J. Greenshaw

**Affiliations:** 1 Faculty of Health Sciences, Department of Psychiatry, University of Alberta, 1E1 Walter Mackenzie Health Sciences Centre (WMC), 8440 112 St NW, Edmonton, AB T6G 2B7 Canada; 2 Research Priorities and Implementation, Research Innovation and Analytics, Alberta Health Services, Calgary, AB Canada; 3 Department of Public Health, Alberta Health Services, Fort Mc Murray, AB Canada; 4 Institute of Health Economics and School of Public Health, University of Alberta, Edmonton, AB Canada

**Keywords:** Depression, Mobile health, Supportive text messages, Intervention, Control

## Abstract

**Background:**

To complement the oversubscribed counselling services in Alberta, the Text4Mood program which delivers daily supportive text messages to subscribers was launched on the 18th of January, 2016. This report presents an evaluation of self-reports of the impact of the program on the mental wellbeing of subscribers.

**Methods:**

An online link to a survey questionnaire was created by an expert group and delivered via text messages to mobile phones of all 4111 active subscribers of the Text4Mood program as of April 11, 2016.

**Results:**

Overall, 894 subscribers answered the survey (overall response rate 21.7 %). The response rate for individual questions varied and is reported alongside the results. Most respondents were female (83 %, *n* = 668), Caucasian (83 %, *n* = 679), and diagnosed with a psychiatric disorder (38 %, *n* = 307), including Depression (25.4 %, *n* = 227) and Anxiety (20 %, *n* = 177). Overall, 52 % (*n* = 461) signed up for Text4Mood to help elevate their mood and 24.5 % (*n* = 219) signed up to help them worry less. Most respondents felt the text messages made them more hopeful about managing issues in their lives (81.7 %, *n* = 588), feel in charge of managing depression and anxiety (76.7 %, *n* = 552), and feel connected to a support system (75.2 %, *n* = 542). The majority of respondents felt Text4Mood improved their overall mental well-being (83.1 %, *n* = 598).

**Conclusion:**

Supportive text messages are a feasible and acceptable way of delivering adjunctive psychological interventions to the general public with mental health problems. Given that text messages are affordable, readily available, and can be delivered to thousands of people simultaneously, they present an opportunity to help close the psychological treatment gap for mental health patients in Alberta and elsewhere.

## Background

Mental disorders can cause significant changes in behaviour, thought patterns, and mood of individuals in all ages and socioeconomic backgrounds [1]. Mental illness is a complex and pervasive burden to both individuals and our health system; its treatment is often challenging and resource intensive [2–4]. Mental health problems vary greatly by their nature, ranging in presentation from insular, episodic problems to longer term, chronic conditions requiring continuous, ongoing therapy. Mental illness is currently the most prevalent disability in Canada, generating 70 % of documented costs with a cumulative annual economic impact of approximately $8B in direct costs (i.e. hospital and physician visits and medications), and between $11B and $50B in indirect costs [5]. Mental illnesses are of primary health, social, and economic significance, given their common coexistence with other mental health and/or other chronic diseases [6, 7]. By 2030, prevalence estimates identify depression to be the leading disability around the globe [8]. Identifying effective, cost-conservative treatments and management techniques for mental illness, especially depression, is a priority for health care systems worldwide.

Depressive and bipolar disorders are the most prominent mental health problems in Canada, with an estimated prevalence of 4.7 and 1.5 %, respectively, with 11.3 and 2.6 % of adults reporting symptoms of major depressive and bipolar disorders at some point in their lives [9]. Women are diagnosed with depressive disorders at approximately double the rate of their male counterparts [10] and are more likely to seek medical attention for this condition. As with many mental health illnesses, depression is a diverse condition, with an equally diverse set of treatment options including pharmacotherapy, psychosocial interventions, counselling, and outreach services [11]. These services usually require referral and are offered only in brick-and-mortar health centres with highly trained health care professionals who can only serve a limited number of clients per day using the traditional therapeutic intervention methods.

The recent Alberta provincial gap analysis revealed that approximately 20 % of adults experienced addiction or mental health problems. Of the surveyed adults, 49 % either needed but did not receive or did not receive adequate services to address their mental health needs. The gap analysis also identified a lack of sufficient access to counselling in 42 % of depressed Albertans. In fact, less than 25 % of depressed Albertans reported their mental health service needs were fully addressed. These findings are consistent with program-level survey reports indicating that mental health service needs exceed available resources [7].

The 2014 gap analysis also revealed significant geographic variations in the distribution of disease burden for addictions or mental health problems, with northern communities experiencing on average the highest prevalence of mental health problems in Alberta. Rural Albertans are disadvantaged with respect to mental health and addictions services access. Given that the most commonly reported treatment modality for addictions or mental health problems is individual therapy and counselling, it is unsurprising that unmet counselling needs are common.

The issue of disparate service access and disproportionate mental health and addictions disease burden in northern populations is further exacerbated by current underutilization of existing mobile and electronic technology to reach clients. Gap analysis findings revealed that only a minority of programs provided screening, assessment, treatment, peer support and follow-up care by phone (between 19 to 40 % depending on the service), and use of the internet is almost negligible for mental health and addictions service delivery (between 2 and 7 % of surveyed service providers) [7].

Mobile health technology offers a convenient, cost conservative, and widely accessible modality for implementing population-level interventions. In particular, the use of text messaging either alone or in conjunction with other interventions has expanded greatly over the past five years in health care [12], and especially in mental health care [13]. A recent global survey revealed a significant increase over the last two years in the proportion of individuals’ median reported use of internet (87 %) and ownership of smart phones (68 %). Among Western countries, Canada is in a high-use group, with 90 % of Canadians reporting use of the internet and 67 % owning a smartphone [14] and within Canada, Albertans were the highest users of wireless phone technology (over 90 % of households) [15]. Within this context, text messaging offers an innovative, cost-effective strategy to enhance mental health services accessibility and improve the efficiency of the overall service delivery [16]. Comparatively, text messaging offers significant advantages over similar technologies (e.g., email or messaging apps) beyond its cost and efficiency. Text messaging is available on most cell phones, and does not require software or mobile application downloads, external equipment, fixed lines, or other hardware to function. Text messages are secure (e.g., can be password protected and stored until accessed by the end user), and the communication process relies on increasingly ubiquitous cell phone infrastructure, accessible even in the most remote regions. As noted by Lim and colleagues, text messaging is a ‘respectful and useful mode of sending a message’ that lacks the marketing and spamming challenges associated with other similar technologies [17, 18].

Despite of high accessibility of mobile technologies [12], their application in the area of mental health and addictions service delivery is relatively limited [7]. However, text messages have been used in multiple areas of mental health including addictions (31 %), schizophrenia (22 %), and affective disorders (17 %), with the majority of interventions employed as supportive messages (42 %) and for self-monitoring (42 %) [13]. Emerging findings document a positive patient attitude towards text messaging as well as improved treatment adherence, symptom surveillance, appointment attendance, and patient satisfaction with management and health care service provisions [13, 19–25]. Text messaging use has also been well-demonstrated in a number of public health interventions [13, 19]. Nonetheless, there are recurring calls for comprehensive evaluative studies quantifying specific benefits, challenges, intervention effects, and cost effectiveness. As noted by Hall and colleagues (2015), there are key features of both the technology and the intervention itself that require further investigation, including the role of frequency and timing of messages, the duration and interactivity level of text message interventions, the relative influence of adjunct interventions and communication modalities, and the influence of design-level use of behaviour change theory on the overall intervention effects [12].

TheText4Mood program evaluation was designed to provide guidance for the further development and refinement of a program to support text-based intervention in the area of mental health and addictions services in Alberta. Patients in Northern Alberta can self-subscribe to the Text4Mood program to receive daily supportive text messages for six months by simply texting the word ‘mood’ to 7606703130. The messages were written by cognitive behaviour therapists and counselors in partnership with mental health patients and are pre-programmed into an online software which delivers the messages at 10.00 h each day. Text messages have been formulated based on cognitive behaviour therapy (CBT) principles to predominantly target mood and anxiety symptom improvement. A different message is sent each day with no repetition of messages throughout the 180-day subscription period. The same message is delivered to all subscribers according to the day they joined the program, with no tailoring of messages to meet individual patient needs.

Examples of the text messages included the following:

- If you keep on going, maintaining your hope and belief that something good will happen, it generally does. One day at a time.
- There are two days in the week we should not worry about, yesterday and tomorrow. That leaves today, live for today.
- What you do today, will determine how you are tomorrow. Rise up and take advantage of whatever opportunities today present and success will be yours tomorrow.
- Today, I will focus on what I have instead of what I have not. Within me lies to power to succeed. I will work with what I have to reach my goals.
- Compete only with yourself, measure your progress by looking backward. Your own hopes and aspirations are the only limits to how far you can achieve.

The Text4Mood program was launched on the 18^th^ of January 2016 by the Alberta Health Services and had 4111 subscribers by the 12^th^ of April 2016.

Given this background, the aim of this study was to evaluate the demographic and clinical profile of subscribers, the impact of a supportive text messaging program on the subscribers’ self-reported mental wellbeing and the attitudes of subscribers towards the mobile mental health intervention.

## Methods

### Study setting and subscribers

Public basic health care is provided universally to all residents of Alberta (4,227,881 [26]) without direct point-of-service payments through a provincial health insurance program administered by the Alberta Health Services. The province is divided into five administrative health care zones: Calgary zone, Edmonton zone, North zone, South zone and Central zone. The Text4Mood program was designed specifically to target residents of the North zone, although residents of other zones were not precluded from signing up for the program. The North zone has the largest geographic area of all Health zones, with a population catchment of 501,367 in 2014 [26]. There are two medium sized cities in the North zone, Fort McMurray and Grande Prairie. Both cities have regional hospitals that also serve the physical and mental health needs of smaller outlying towns and cities, some of which are hundreds of kilometres away. The vast majority of the region is rural-remote, with smaller primary healthcare facilities in towns and cities. Given the geographic spread, residents in these towns usually travel long distances to either Fort McMurray or Grande Prairie for secondary and specialised healthcare services (including Cognitive Behaviour Therapy), which often impairs the practical accessibility of these services. The Text4Mood program was therefore designed and advertised primarily to patients seeking psychological or counselling services for depression and anxiety at primarily and secondary care centres across Northern Alberta, in order to help narrow the psychological treatment gap for patients experiencing these mental health difficulties. However, as patients self-subscribe to the program, there is no mechanism to prevent patients with other mental health conditions or even the general public from subscribing to the service. All survey data, including diagnostic information was self-reported by subscribers and not validated using clinical assessment tools (e.g., rating scales or structured clinical interviews).

### Developing and administering the survey tool

The survey questions were formulated based on the objectives of the study and available evidence from peer-reviewed literature. The survey consisted of predominately Likert scale responses and a few open-ended short answer questions evaluating the following:

#### Sociodemographic characteristics

(i) Sex, (ii) Age, (iii) Ethnic background, (iv) Educational background, and (v) Employment status.

#### Clinical characteristics

- Whether respondents have been diagnosed with a mental health condition or not.
- If diagnosed with a mental condition, what was the condition?
- Whether respondents have been diagnosed with a physical health condition or not.
- If diagnosed with a physical health condition, what was the condition?

#### How and why respondents subscribed to the Text4Mood program

- Where did the respondents hear about the Text4Mood program?
- Why did the respondents sign up for the Text4Mood program?
- Did respondents need any assistance in signing up for the program?

#### Subscribers’ response to and perceptions about the supportive text messages

- How frequently did the respondents read the daily supportive text messages?
- How did the respondents respond to the supportive text messages?
- Did the respondents perceive the supportive text messages to be on-topic, to the point, supportive, and positive?

#### Impact of the Text4Mood program of subscribers’ mental well being

- Agreement or disagreement with various statements reflecting personal positive benefits of the Text4Mood program on participants’ mental wellbeing

#### Subscriber satisfaction and interest in technologically based health services

- How satisfied were respondents with the frequency of the supportive text messages?
- Ideally, how frequently will respondents like to receive the supportive text messages?
- Will participants welcome other technologically delivered healthcare solutions as part of their overall health care packages?

Draft survey questions were programmed into Survey Select, an online survey tool owned and operated by the Alberta Health Services Evaluation Services Team. The online survey was pre-tested with ten respondents (four of whom were clinically depressed) who were not enrolled in the Text4Mood program. The survey questions were revised based on pre-test findings. Additional Likert scale responses were drafted from several of the open-ended questions. A second survey pretest was undertaken on five additional respondents (two of whom were clinically depressed) and took participants approximately 5 min to complete. Incentives were not offered to respondents for taking part in the survey pretests, nor as part of the survey evaluation itself. Participation in all of the questionnaires was entirely voluntary.

The online survey link was distributed to all subscribers of the Text4Mood program on their 42^nd^ day (six weeks) of the program. The link was sent to subscribers between the 1^st^ of March 2016 and the 11^th^ of April 2016 to allow all subscribers reaching day 42 of the program to complete the survey. On the final day of the program, a reminder text message with the survey link was sent to all 4111 subscribers of the Text4Mood program, including those who had not yet reached six weeks on the program, encouraging them to complete the survey. The survey was left open for 24 h after that and closed on the 12^th^ of April 2016.

### Data analysis

All data from the survey tool was recorded in a spread sheet. Quantitative data was analysed using descriptive statistics. Results were reported as either numbers and percentages or bar graphs. Qualitative data was analysed thematically and representative verbatim quotes provided as appropriate to support the quantitative results.

## Results

Of 4111 active subscribers to the Text4Mood program, 894 completed the survey, amounting to an overall response rate of 21.7 %. The demographic and clinical characteristics of the respondents are summarised in Table 1. The ‘n’ for each individual characteristics varied and is reported alongside the results.

**Table 1:** Demographic and clinical characteristics of respondents

Variable		*N* (%)
Gender^a^	Male	142 (17.5 %)
	Female	668 (82.5 %)
Age^b^	≤15	16 (2 %)
	16–25	122 (15.1 %)
	26–45	410 (50.7 %)
	46–65	253 (31.3 %)
	≥65	7 (0.9 %)
Ethnic origin^c^	Caucasian	679 (83.1 %)
	First Nations/Metis/Inuit	42 (5.1 %)
	Asian	32 (3.9 %)
	Black	8 (1 %)
	Other	40 (4.9 %)
	Prefer not to disclose	16 (2 %)
Highest Education^d^	Post Graduate Degree (MSc, PhD)	98 (12 %)
	University Degree or Diploma	260 (32 %)
	College Diploma	189 (23 %)
	Apprenticeship/Trade Certificate or Diploma	57 (7 %)
	High School Diploma	161 (20 %)
	Junior High School	24 (3 %)
	Elementary School	0 (0 %)
	Prefer not to disclose	16 (2 %)
Employment status^e^	Employed Fulltime	441 (49 %)
	Employed Part-time	151 (17 %)
	Self-employed	54 (6 %)
	Student	89 (10 %)
	Homemaker	45 (5 %)
	Unemployed with no income	45 (5 %)
	Unemployed on Government social benefits	28 (3 %)
	Retired	15 (1.7 %)
	Prefer not to disclose	19 (2.1 %)
Mental health condition respondents had been diagnosed with^e,f^	Depressive Disorder	227 (24.5 %)
	Anxiety Disorder	117 (19.8 %)
	Trauma and stressor related disorder	58 (6.5 %)
	Bipolar Disorder	34 (3.8 %)
	Substance Related Disorder	24 (2.7 %)
	Personality Disorder	14 (1.6 %)
	Schizophrenia Spectrum Disorder	9 (1 %)
	Other Disorders	19 (2.1 %)
	None	454 (56 %)
Where respondents receive medical care^e^	Edmonton Zone	375 (42 %)
	North Zone	301 (34 %)
	Calgary Zone	114 (12.8 %)
	Central Zone	51 (5.7 %)
	South Zone	19 (2.1 %)
	Outside Alberta	33 (3.7 %)

^a^Total *N* = 810 ^b^Total *N* = 808 ^c^Total *N* = 817 ^d^Total *N* = 814 ^e^Total *N* = 893

^f^Participants were able to select more than one answer; sum percentages is greater than 100 %

The majority of respondents were of female sex (83 %, *n* = 668) and Caucasian ethnicity (83 %, *n* = 679). Half of the respondents were 26–45 years old (51 %, *n* = 410), while almost one-third were 45–65 years old (31 %, *n* = 253). Many respondents identified university degree or diploma (Bachelor’s) as their highest level of education (32 %, *n* = 260), followed by college diploma (23 %, *n* = 189), high school (20 %, *n* = 161), Post-Graduate Degree (12 %, *n* = 98), and Apprentice or Trade certificate (7 %, *n* = 57). Overall, 38 % (*n* = 307) of participants reported they have been officially diagnosed with a chronic mental health condition including depressive disorder (25.4 %, *n* = 227) and anxiety disorder (20 %, *n* = 177). The majority of respondents received medical care from the Edmonton zone (42 %, *n* = 375) and the North zone (34 %, *n* = 301).

### How respondents’ learnt about the Text4Mood program and reasons for subscribing to the program

The most common ways respondents heard about the Text4Mood program were through a friend (22 %, *n* = 198), on the news (19 %, *n* = 168), other (18 %, *n* = 161), on a website (14 %, *n* = 125), on Facebook (11 %, *n* = 98) and from a poster (9 %, *n* = 80). The least common means of hearing about the program were from a poster (6 %, *n* = 60), from a clinic (3 %, *n* = 30), the participant’s doctor or nurse (3 %, *n* = 31), and Twitter (2 %, *n* = 20).

When respondents were asked why they signed up for the Text4Mood program, some respondents selected multipole responses, with approximately half of respondents indicating they signed up for text for mood to help elevate their mood (51.6 %, *n* = 461) or to help them feel better (49 %, *n* = 440). About a quarter indicated that they signed up for the program to help them worry less (24.5 %, *n* = 219) whilst about a fifth said they signed up because of the novelty or fun of the program (19.7 %, *n* = 176). Furthermore, 15.9 % of respondents indicated they signed up for the program for ‘other reasons’. Of those respondents who selected the ‘other’ category, the majority of the respondents signed up for this program to get support, motivation, inspiration/encouragement, to exercise positive thinking or to use the program as a reminder to practice mindfulness and view life from a positive perspective. In addition, a few respondents signed up to address specific concerns such as, “ground my anxiety,” help with “potential postpartum depression,” or “to help fight an addiction.” Other respondents signed up to be able to share positive thoughts or to support their families or friends who are dealing with mental health issues. A few respondents were curious about the service and wanted to try it. Finally, some respondents were mental health service managers or providers (such as psychologists) who signed up to test if the service was appropriate to recommend to their clients or their clinical staff.

*“I work in mental health and wanted to see what the service was like prior to recommending to my clients.”*

*“To see if my patients would benefit from it”*

The majority of respondents did not need assistance signing up for the program (96 %, *n* = 694).

### Subscribers’ response to and perceptions of the supportive text messages

When asked how often they read the text messages, the majority of respondents (90 %, *n* = 648) said they ‘always’ read the text messages. Only 8.1 % (*n* = 58) read the text messages ‘often’ and 1.5 % read the messages ‘sometimes’.

Overall, three-quarters of respondents (76 %, *n* = 545) said that they ‘always’ understood the text messages that they received, 22.9 % said they ‘mostly’ understood the text messages, and 1.4 % said they ‘sometimes’ understood the text messages. The actions respondents reportedly take in response to the daily text messages are:

- “I read and reflect on the messages”- (65 %, *n* =584)
- “I return to read the text messages for support more than once”- (33 %, *n* = 295)
- “I read the messages and take a positive action”- (29 %, *n* = 258)
- “I read the text messages and take no action”- (18 %, *n* = 160)
- “I read the messages and take a negative action”- (0.1 %, *n* = 1)
- “I do not read the text messages”-(0.9 %, *N* = 8)

**Fig. 1:**
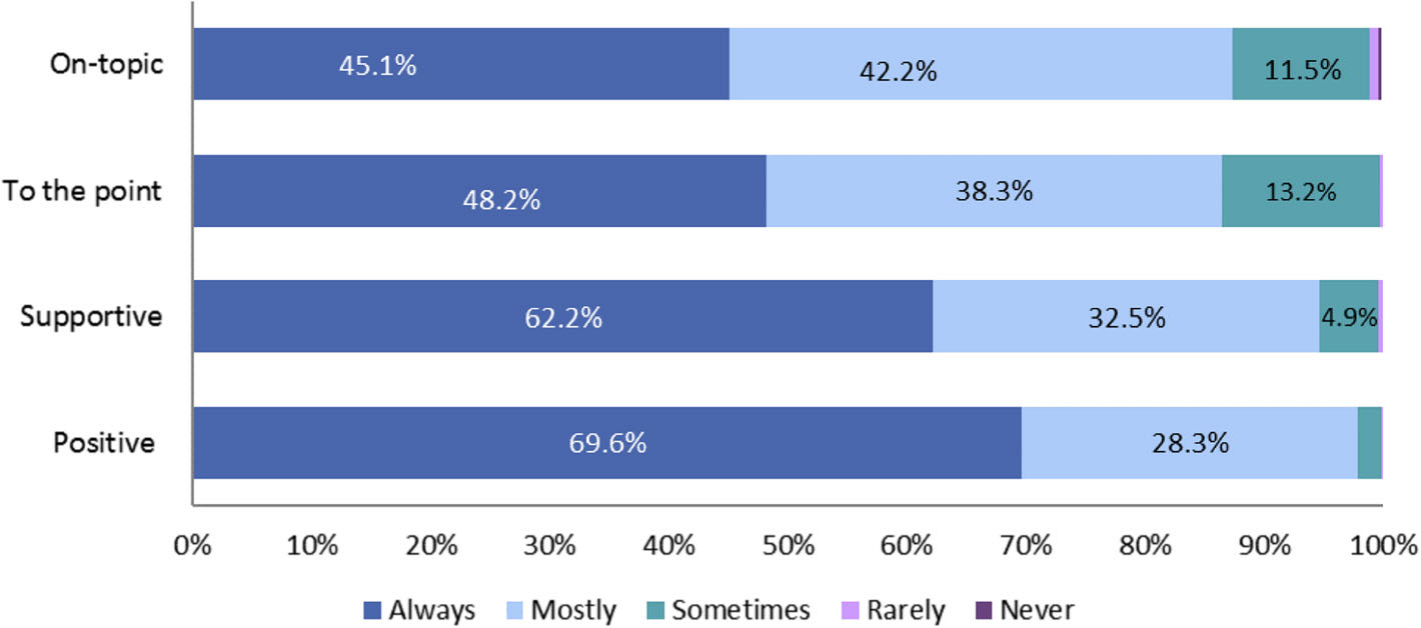
Respondent’s Perception of the Text Messages, *n* = 720

Using a Likert scale, participants were asked to indicate their perceptions about the contents of the supportive text messages as to whether they were on-topic, to the point, supportive, and positive. The responses are as summarised in Fig. 1.

Overall, respondents always found the Text4Mood messages to be positive (69 %, *n* = 501) and on-topic (45 %, *n* = 325). Respondents identified messages as always supportive 62 % (*n* = 448) of the time and always to the point 48 % of the time (*n* = 347).

### Impact of the Text4Mood program on subscribers’ mental well being

**Fig. 2:**
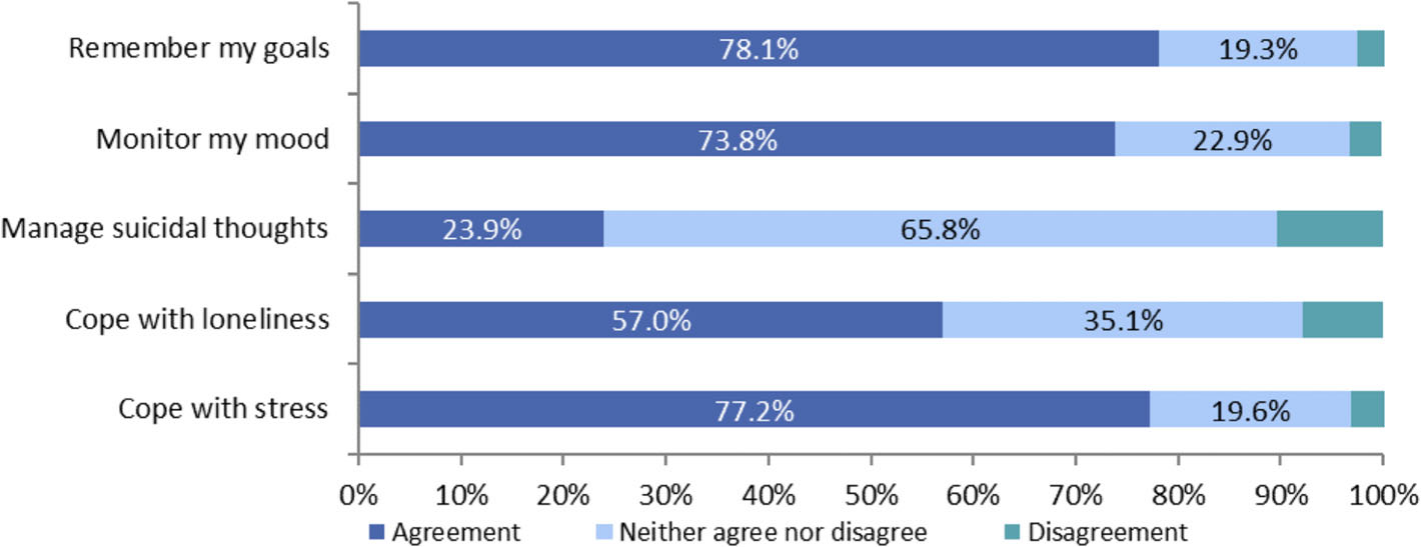
Benefits of Text4Mood I, *n* = 720

**Fig. 3:**
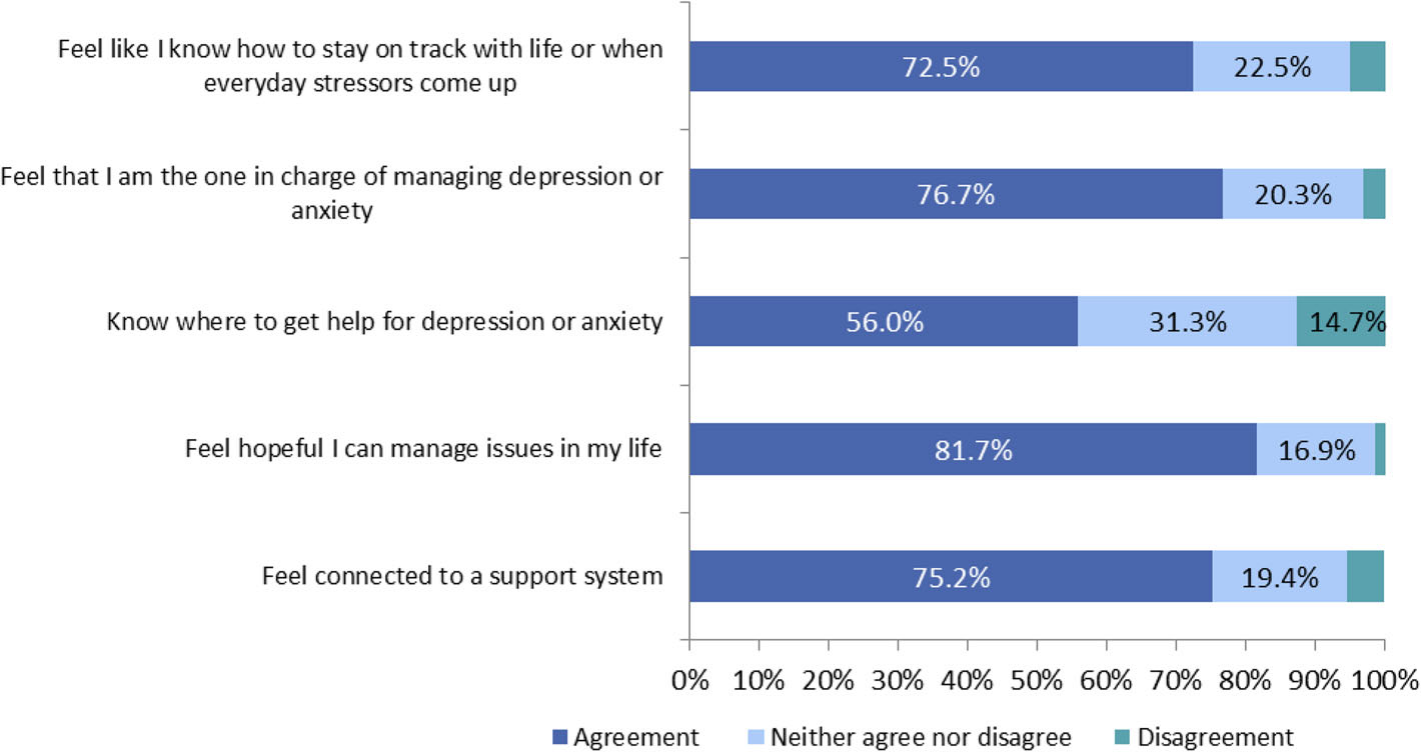
Benefits of Text4Mood II, *n* = 720

**Fig. 4:**
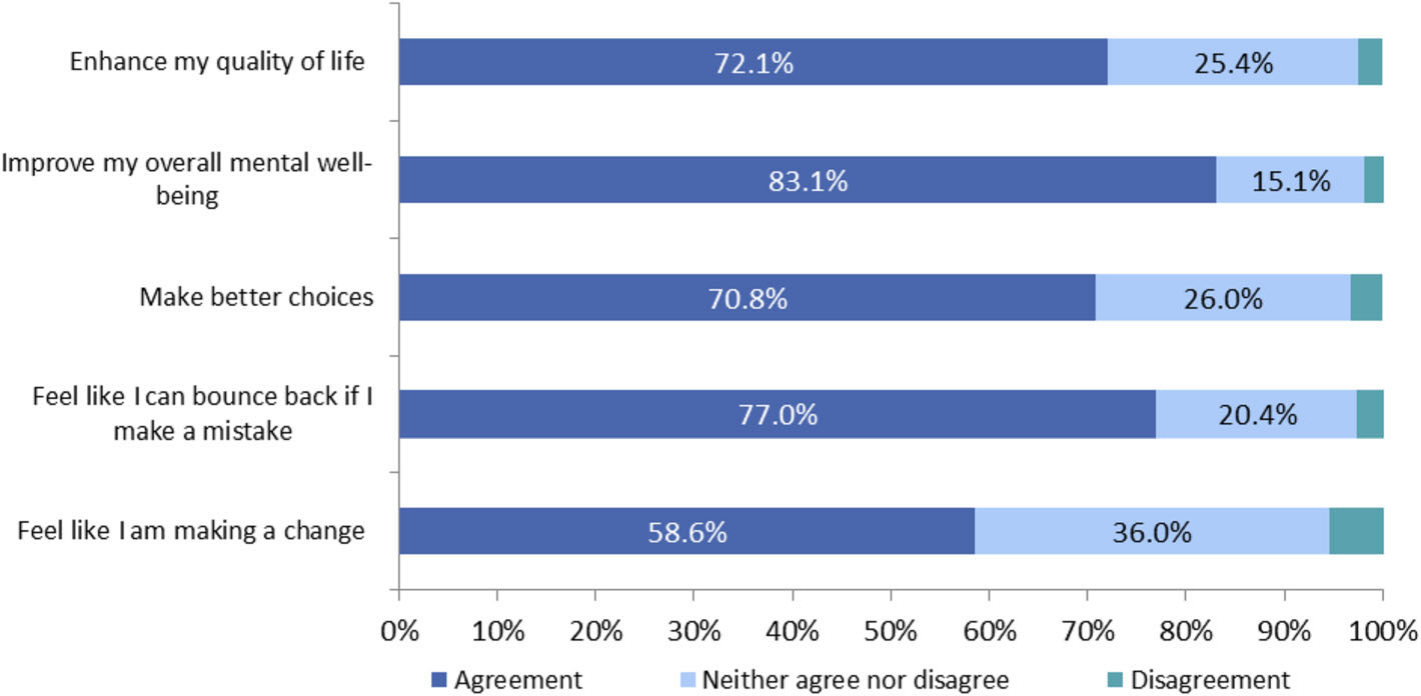
Benefits of Text4Mood III, *n* = 720

Participants were asked to express agreement or disagreement with statements reflecting the positive benefits of the daily supportive text messages to them on a personal level. The responses are as summarized in Figs. 2, 3 and 4.

In each category, the majority of respondents were in agreement with each statement. Most respondents felt hopeful in managing issues in their lives (81.7 %, *n* = 588), followed by feeling in charge of managing depression and anxiety (76.7 %, *n* = 552) and then feeling connected to a support system (75.2 %, *n* = 542).

Most respondents felt hopeful in managing issues in their lives (82 %, *n* = 587), followed by feeling on track with life or when everyday stressors come up (73 %, *n* = 522).

The majority of respondents felt the Text4Mood improved their overall mental well-being (83.1 %, *n* = 598) and made them feel like they could bounce back if they made a mistake (77 %, *n* = 554).

Furthermore, 75 % of respondents (*n* = 545) said that the messages made them feel ‘supported’ whilst only 6.5 % indicated that the messages ‘Made no difference’. Only 1.1 % indicated that the messages made them ‘Annoyed’. The majority of the respondents who chose ‘other’ (18 %, *n* = 127) stated that receiving the daily text message made them feel good. They found the text messages set a positive tone for their morning, making them feel “motivated,” “happy,” and “inspired.” Some mentioned that they share them with friends and families to spread the inspiration and thought.

*“…they are so positive and uplifting everyone should be having access to this wonderful tool.”*

*“When everything is going bad, that message brightens my day. I look forward to it every day.”*

*“They just help me to get or stay more positive when I am struggling.”*

Some respondents highlighted that they had mixed feelings about the text messages. They found some messages to be inspiring and supportive, while other messages were ineffective and frustrating. Some respondents found that the text messages did not alter their mood or feelings in anyway. A few participants found messages that had a religious tone (e.g. talked about God) to be inappropriate. Others felt that the messages reminded them of their personal challenges leaving them discouraged.

*“I am grateful for them, and basically after reading it, I tucked it away in my notes. Sometimes they made a LOT of sense for that particular day/moment, but I do not really feel like they, in and of themselves saved me. They are like a treat each day, and some days I share them, some days they really hit home and other days they made me feel upset, anxious and guilty. But I DO NOT want to stop getting them!!”*

*“Some are good. Others don't seem to be appropriate for what I thought the service is.”*

### Subscriber satisfaction and interest in technologically based health services

Overall, the majority of respondents were very satisfied or satisfied with the frequency of text messages received (95 %, *n* = 681). Four percent of respondents (*n* = 32) said they were neither satisfied nor dissatisfied with the frequency of the text messages, whilst only 1 % (*n* = 8) of respondents were dissatisfied. On the other hand, when asked what should be the ideal frequency with which the Text4Mood program should send messages to subscribers, while the majority of respondents preferred to receive supportive text messages once per day (68 %, *n* = 491), a considerable number preferred to receive the supportive text messages twice daily (17 %, *n* = 122). On the other hand, 12 % (*n* = 86) preferred to receive the messages once every other day and 3 % (*n* = 22) preferred to receive the supportive text messages once a week.

**Fig. 5:**
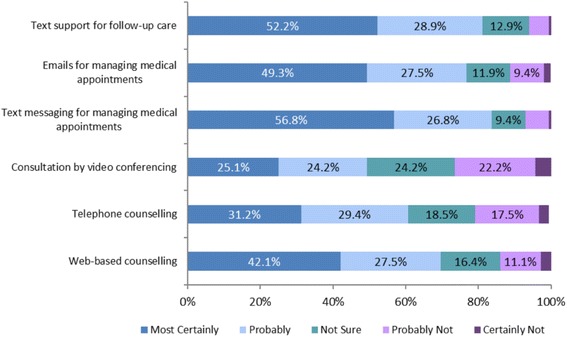
Opinions About Use of Technology-based Services as part of Healthcare, *n* = 720

Respondents were asked if they would welcome a range of technology based services as part of their overall health care packages. The responses are summarized in Fig. 5.

The majority of respondents were most certainly in favor of text support for follow-up care (52 %, *n* = 376) and in favour of text messaging for managing medical appointments (57 %, *n* = 409). Use of technology-based services as part of healthcare was also identified as most certain for Emails for managing medical appointments (49 %, *n* = 355) and web-based counselling (42 %, *n* = 376) by plurality of the respondents.

## Discussion

Overall, both the proportions of females and different types of mental health disorders self-reported by Text4Mood survey respondents were generally consistent with global and provincial mental health prevalence trends [7], and the high, positive subscriber perception of the text messaging intervention was also consistent with a recent review of the use of text messaging in mental health [13].

This study generated unique findings with respect to self-reported rationale for subscribing to a supportive text message service. While most studies report reasons for adults seeking mental health services associated with specific diagnostic conditions (e.g. depression, anxiety), our survey studied both structured and open-ended responses in an effort to understand the underlying rationale for the mobile health service subscription. This approach provided rich data regarding the breadth of symptoms and reasons for seeking text message support. Open-ended findings contribute valuable insights for designing more user-friendly text message support programs that are patient-oriented and can be specifically tailored to patients’ needs.

A recent systematic review [13] emphasized the need for further refinement in greater depth of reporting of the intervention characteristics and their consistent implementation through comprehensive trials. Despite its low response rate, our study attempted to capture facets of implementation quality by integrating evaluative criteria pertaining to fidelity (the degree to which text messages were used as they were intended), dosage (intervention frequency), participant responsiveness (the extent to which Text4Mood stimulated and held subscriber interest across the intervention period), and program reach (participation rates, program scope). For example, the survey tool asked respondents to report on the degree to which text messages were consistently read, and the extent to which they felt they understood the text messages. Overall, respondents reported being very satisfied or satisfied with the frequency of text messages, which may at least in part, help to address some concerns about ethical considerations arising from the use of supportive text messaging in populations with mental health problems raised by Berrouiguet and colleagues (2016). However, we note that these findings should be interpreted with caution given the low survey response rate. Most subscribers were also open to considering the use of text messaging for follow-up care and for managing medical appointments - in both cases to a greater extent than either email or web-based counselling.

Our survey also intentionally solicited reflections from subscribers about whether action planning processes took place as a result of reading the text messages (e.g., reflection, positive action). These are important findings to consider within the context of behaviour change since both reflective and impulsive processes are operating in parallel [27]. While reflective processes (i.e. reading and reflection on the text) may help individuals develop intentionality, important gaps between intention and action have been identified in the literature and have prompted subsequent study into action and coping planning in particular [28–30]. From a behavioural change perspective, text message intervention which can stimulate both action and coping planning is a key design-level objective which should be considered as part of future program development and text message content optimization. This might be particularly important for health support programs in which transition to optimal ‘target behaviour’ is often the goal. Addition of interactive features into the text messaging intervention might further help to advance our knowledge about the impact of the intervention and the integration of behaviour change theory into the actual implementation process [12].

This study captured subscriber perception of mental well-being, with the majority (>75 %) of respondents reporting feeling hopeful about their management of life issues, feeling in charge of managing depression and anxiety, and feeling connected to their support systems. Many respondents reported that the text messaging intervention helped them improve their overall mental well-being (83.1 %) and gave them a feeling that they could ‘bounce back’ if they made a mistake (77 %). In contrast, in a study which examined the usability of the Internet-based component of Breathe (a CBT program designed for adolescents with mild to moderate anxiety and impairments) participants described a much more diverse attitudes toward this eHealth intervention ranging from negative (in 75 % young people) to positive (in 60 % clinicians but only 25 % young people) [31]. While our study does not compare outcomes for the Text4Mood program with CBT, in economic terms, it cost only 3 cents to send a supportive text message to a patient, compared to the cost of a session of CBT which ranges from 100–200 US Dollars in Alberta. Consequently, it will cost only 5.40 US dollars to deliver daily supportive messages to patients for 6 months, compared 1200–2400 US Dollars for 12 sessions of CBT over a six months period. Furthermore, supportive text massages do not require any human resource and messages can be delivered to thousands of patients simultaneously, compared to CBT which is human resource intensive, often with patients having to wait several weeks or months to receive their first session. This potential economic benefit notwithstanding, both the Text4Mood program and our survey are not adequately designed/powered to determine positive/negative effect of the intervention. Therefore, despite the potential cost saving implications with a Tex4Mood program, definitive cost effectiveness comparisons with CBT cannot be made on the basis of our findings.

The Text4Mood text message content was developed from a patient-provider cognitive-behavioural therapy perspective. The integration of theory and patient needs/preferences through pilot testing in the development of text messages in the Text4Mood initiative addressed directly, the question of text message content raised by Berrouiguet and colleagues in their recent systematic review of mobile phone and web-based text messaging in mental health [13]. Readability of the text messages was most likely not a significant issue since almost all respondents (98.9 %) indicated they ‘always’ and ‘mostly’ understood the text messages. Nonetheless, data validation, design and usability testing may be required to assess and further optimize specific messages to enhance their readability and/or comprehension. Furthermore, some respondents reported having ‘mixed feelings’ about the content of the texts. Usability testing of individual responses to text message content is an area that might be considered for future analysis and could make an important contribution to the literature [13].

Finally, with further study, the use of supportive text messages in subpopulations within a provincial health service has the potential to advance knowledge about important differentiating features among therapeutic groups, and may help contribute evidence that addresses potentially important differences in age, gender, educational attainment, and socioeconomic patterns [13]. Emergent findings summarized here will help guide the development and refinement of the Text4Mood program as well as support a provincial business case for an ongoing supportive text messaging program delivery within a large Canadian health service context.

### Limitations, strengths and research implications

A major limitation of this study was the low survey response rate (21 %); it is possible that subscribers who did not participate in the survey might have given different responses. However, the response rate for our survey was greater than those achieved by comparable online surveys [32–34]. Secondly, while the online survey tool was well researched to answer the objectives of the study, it was not a validated instrument, may not have accurately measured our objectives, and the findings are self-reported subscriber perceptions that have not been validated. Our sample was not randomised, thus there was no comparison group to assist with ascertaining the beneficial or detrimental impact of the intervention. Furthermore, we did not test the frequency or duration of text messages to determine the optimal delivery frequency and timing. This is an aspect that should be designed into a subsequent program roll out. In addition, individual-level matched responses would be helpful to examine whether we can match factors to responses in the analysis also at the individual level and compare these with the broader group level responses. Finally, because the survey was completed anonymously, it is possible that some respondents may have completed the survey more than once.

A major strength of our study is that it is the first study to evaluate the feasibility and perceived acceptability of an innovative program using supportive text message to bridge the psychological treatment gap at a population level. Another strength is that the administration of the survey tool and the analysis of the data were conducted independent of the research team by the impartial government Alberta Health Services Evaluations Team, thus eliminating any potential researcher bias in the analysis.

Important issues that require further research include; the optimum frequency and duration for using the Text4Mood program to support recovery from specific mental health conditions as well as to mitigate risk in mental health and considerations about linking the program to geographical and specific mental health services and programs. Furthermore, the potential of current technologies to personalize and tailor messages using smartphones apps which incorporate both subjective and objective information should be explored.

## Conclusions

This study revealed novel findings relating to the underlying rationale for individuals to subscribe to supportive text messaging service, the intervention itself (message content, frequency), and its implementation fidelity, dosage, responsiveness and reach. The study generated important insights about the potential value of supportive text messages for action and coping planning and also described subscribers’ subjective, self-reported belief that supportive text messages improved hope, ability to manage anxiety and depression, improve their perception of connectedness, as well as improve overall mental well-being, and resiliency. Our findings suggest that supportive text messages may be a feasible and acceptable method of delivering psychological treatments to mental health patients, however, further prospective, randomized studies are required to rigorously ascertain the potential positive and negative impacts of the text messaging intervention. Given that text messages are affordable, readily available, and can be delivered to thousands of people simultaneously, they present a potential opportunity to help close the psychological treatment gap for mental health patients in Alberta.
